# Responsive photonic nanopixels with hybrid scatterers

**DOI:** 10.1515/nanoph-2021-0806

**Published:** 2022-03-21

**Authors:** Jang-Hwan Han, Doeun Kim, Juhwan Kim, Gyurin Kim, Ji Tae Kim, Hyeon-Ho Jeong

**Affiliations:** School of Electrical Engineering and Computer Science, Gwangju Institute of Science and Technology, 61005 Gwangju, Republic of Korea; Department of Mechanical Engineering, The University of Hong Kong, Pokfulam Road, Hong Kong, China

**Keywords:** active plasmonics, hybrid scatterers, metamaterials, nanopixels

## Abstract

Metallic and dielectric nanoscatterers are optical pigments that offer rich resonating coloration in the subwavelength regime with prolonged material consistency. Recent advances in responsive materials, whose mechanical shapes and optical properties can change in response to stimuli, expand the scope of scattering-based colorations from static to active. Thus, active color-changing pixels are achieved with extremely high spatial resolution, in conjunction with various responsive polymers and phase-change materials. This review discusses recent progress in developing such responsive photonic nanopixels, ranging from electrochromic to other color-changing concepts. We describe what parameters permit modulation of the scattering colors and highlight superior functional devices. Potential fields of application focusing on imaging devices, including active full-color printing and flexible displays, information encryption, anticounterfeiting, and active holograms, are also discussed.

## 1 Introduction

Pixels of a display are addressable color elements in a raster image plane [1]. Three pixels are often configured as a primary set to generate full color across the whole visible regime through the combinations of three colors, namely red, green, and blue (RGB) [2], [3], [4]. Light-emitting diodes operating at a small scale, called micro/nano light-emitting diodes (LEDs), are the most representative examples and work on the basis of electroluminescence; i.e., a process of energy conversion from the electron to the photon [2, 5]. Micro/nano LEDs currently lead the display market owing to their small structural dimensions and thus high spatial resolution [5], [6], [7], and have possible applications ranging from flexible displays to color-changing clothes and large-scale wallpaper [6, 8, 9]. However, the high spatial resolution of the LEDs means that extremely small-scale electrical circuits and complex integration are essential [2, 10, 11]. Furthermore, the outdoor operation of the LEDs under sunlight remains in its infancy due to the low quantum efficiency of the LEDs [3], [4], [5, 12].

A promising alternative means by which to address such challenges is the use of photonic nanopixels, comprising nanoscale photonic elements with an active medium or material [13], [14], [15], [16]. The main point of difference of the photonic version from the conventional electroluminescence-based pixels is the unique optical coloration resulting from strong, resonant light–matter interactions in response to light [17], [18], [19], [20], [21], [22]. All colors are thus generated from external photons, and the pixels work perfectly under sunlight with no electrical circuit. In particular, various strong light–matter interactions can occur depending on the class of materials and the geometrical dimensions of the nanostructures. For instance, compound semiconductors smaller than several tens of nanometers, known as quantum dots, can convert the energy state of a single-wavelength light source (i.e., color down- or up-conversion), often a laser or LED, according to their bandgap energy and form [23], [24], [25], [26]. However, due to their tiny size and complex material composition, most quantum dots are chemically synthesized, which is often incompatible with complementary metal–oxide–semiconductor (CMOS) techniques [27, 28]. Additionally, they typically consist of toxic materials (e.g., Cd, Se) and chemically unstable [27, 29], [30], [31].

In contrast, metallic (10–100 nm in size) and dielectric nanoparticles (with size on the order of the wavelength of light) have dimensions that are nanolithographically accessible with CMOS compatible materials, and they show unique solid resonant scattering colors upon the irradiation of multiwavelength light, typically white light [17], [18], [19]. These scatterings stem from the strong optical near-field enhancement of surrounding nanoparticles through the collective motions of electrons (i.e., free electrons in metals and bound electrons in dielectrics), known as plasmonic [17, 18] and Mie resonances [19, 20]. The control over the material composition [32], [33], [34], [35], structural geometry [33, 36], [37], [38], [39], [40], [41], and overall arrangement within an ensemble [34, 42, 43] or array [42, 44], [45], [46] of the nanostructures determines the nature of the plasmonic and Mie scattering colorations. Crucially, active scattered colors can be obtained when an applied stimulus controls one of these parameters. The use of responsive materials, including phase-change materials and responsive polymers, makes plasmonic nanoparticles function as small optical on/off switches and color-tunable pixels.

We highlight recent developments in responsive plasmonic and Mie resonant-based photonic nanopixels driven by external stimuli. In conjunction with responsive materials, we discuss how one of the intrinsic parameters that determine the resonant color of the nanostructures can be tunable, allowing the realization of active color-changing switches/pixels at the single-nanoparticle level. Such nanopixels are categorized and discussed according to their dynamic mechanisms and types of input stimulus (e.g., electrical signal, light, heat, strain, gas). We also highlight the emerging light-conversion systems based on combinations of nanoscatterers with functional materials (e.g., quantum dots, upconversion nanoparticles, and transition metal chalcogenides). Additionally, potential applications, including flexible displays, encryption, and active holograms, are highlighted.

## 2 Nanophotonic scatterers

**Figure 1:**
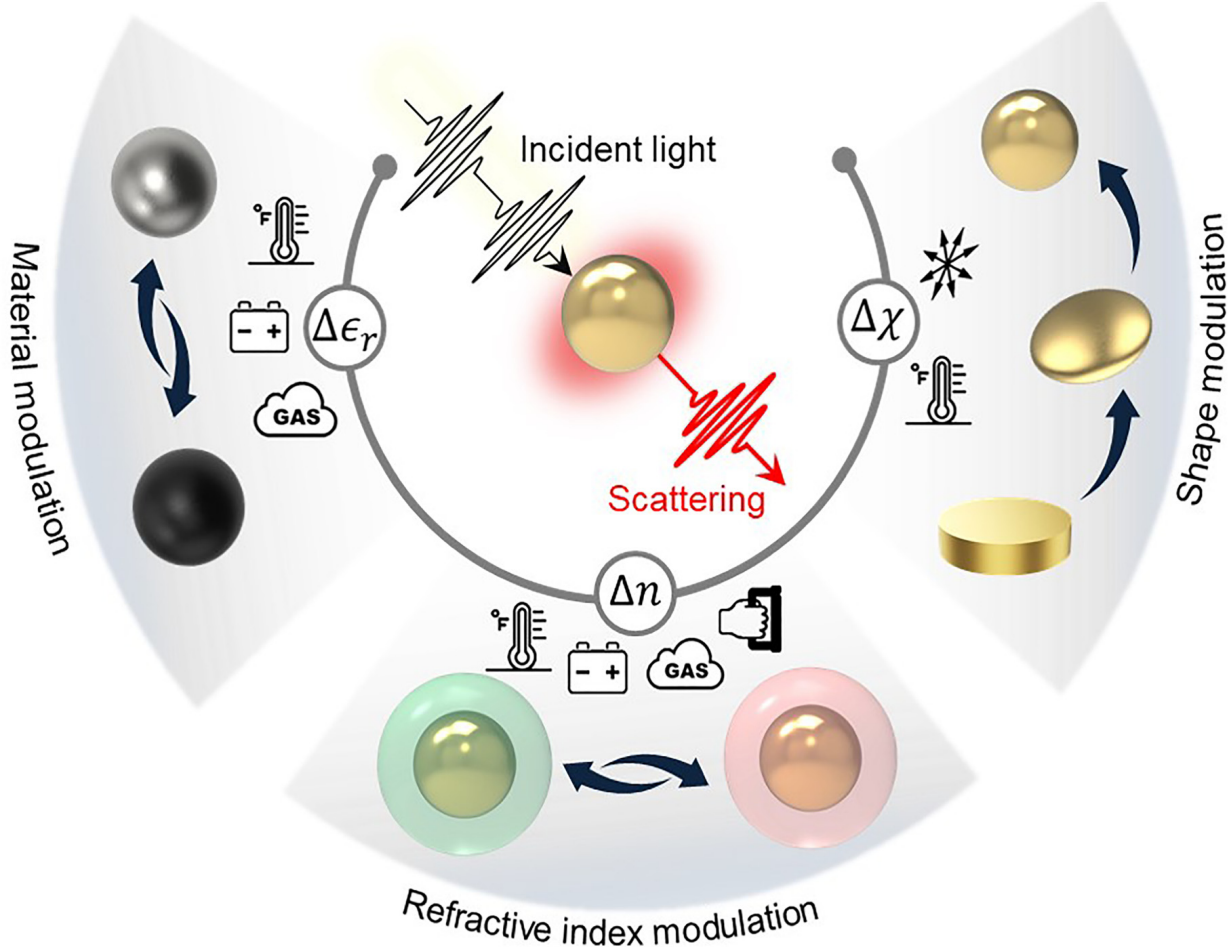
Three modulated parameters in the engineering of active coloration with plasmonic and Mie resonance nanoscatterers: the real part of the complex dielectric constant $\left(\Delta \epsilon_{r}\right)$ and shape factor (Δχ) of the nanoparticle and the refractive index of the surrounds of the nanoparticle (Δn) .

Photonic nanopixels are color-changing nanoparticles and structures in the subwavelength regime that respond to an external stimulus, as shown in Figure 1. Two components are essentially required for this response, namely (i) a photonic pigment having nanoscale dimensions but interacting at the wavelength of light and (ii) an active medium or material that modulates scattering colors. The former has two physical mechanisms of activity, namely plasmonic and Mie resonances. The combinations between the photonic nanopigments with an active medium acting as photonic nanopixels are discussed in the following sections. A variety of responsive photonic nanopixels are categorized depending on three modulated parameters, i.e., the real part of the complex dielectric constant $\left(\Delta \epsilon_{r}\right)$ and shape factor (Δχ) of the nanoparticle and the refractive index of the surrounds of the nanoparticle (Δn) .

### 2.1 Plasmonic nanoscatterers

**Figure 2:**
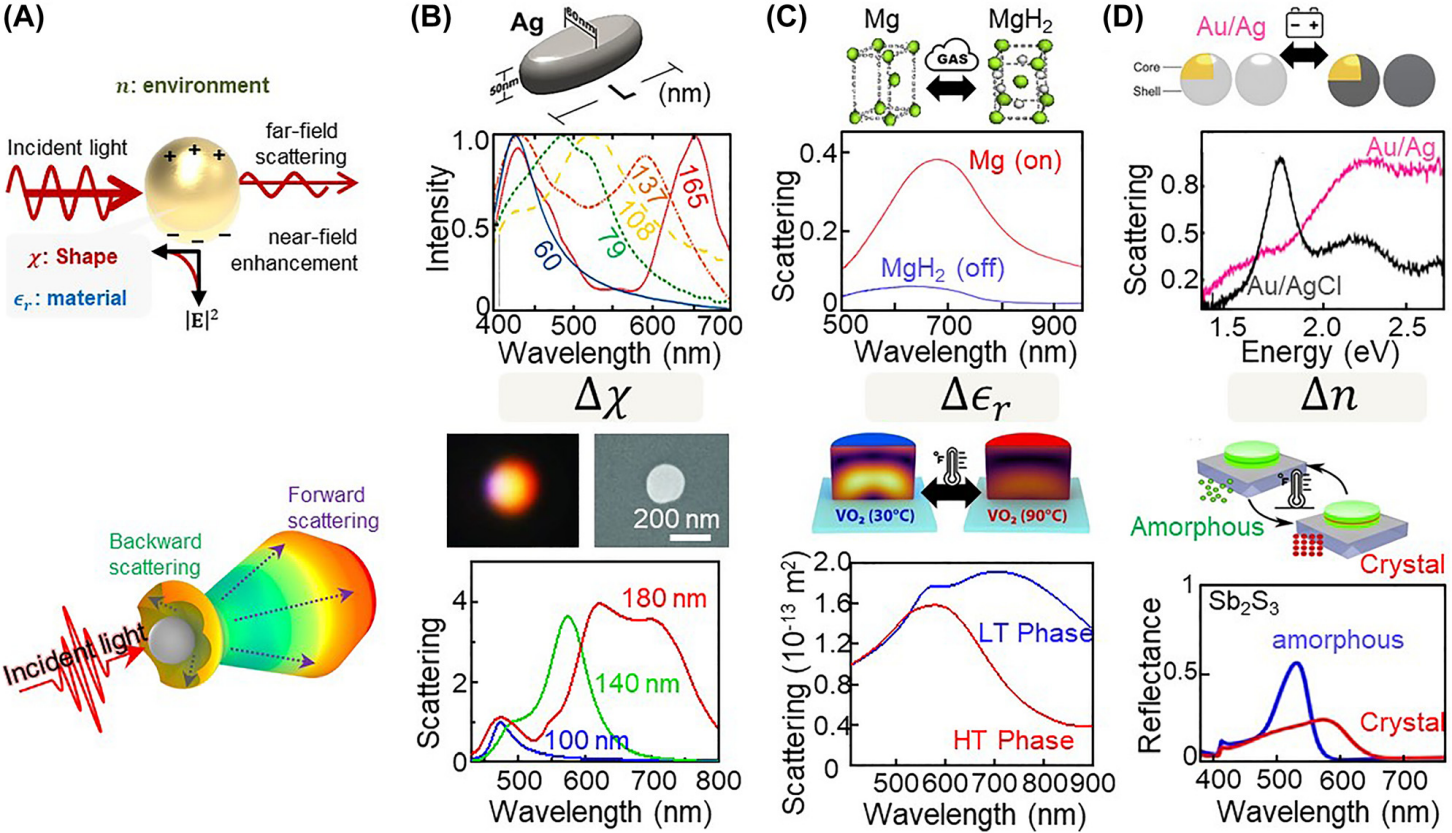
Plasmonic (top panel) and Mie (bottom panel) resonances: (A) associated schematics of the optical interactions and colorations due to changes in the nanoparticle’s (B) shape and size (reproduced with permission [19, 77]. Copyright 2016, American Association for the Advancement of Science and 2014, Europe PMC), (C) material composition (reproduced with permission [78], [79], [80], Copyright 2018, American Association for the Advancement of Science, 2009, Elsevier B.V., and 2021, American Chemical Society), and (D) surrounding optical environment (reproduced with permission [81, 82]. Copyright 2015, American Association for the Advancement of Science, and 2020, Optica Publishing Group).

Localized surface plasmonic resonance (LSPR) is a strong resonant light–matter interaction (top panel in Figure 2A), which occurs when light illuminates a metallic nanoparticle or structure whose diameter is smaller than the light’s wavelength, typically in the range of 10–100 nm [17, 18, 47]. If the nanoparticle and structure are smaller than 10 nm, then the mean free path of the free electrons can be longer than the dimension of the nanoparticle, resulting in quantum effects [48, 49]. Meanwhile, nanostructures larger than 100 nm often give rise to undesired higher-order resonance modes and radiation damping effects [47, 50]. This means that at least one dimension of the nanostructure needs to be larger than 10 nm and smaller than a few hundred nanometers to permit the excitation of LSPR. Otherwise, exceptions are possible when the design parameters (i.e., the nanoparticle’s structural geometry and material composition) are carefully optimized for higher-order resonance features, although several challenges, such as a wide spectral bandwidth (low color purity), multiple peaks (color superposition), and low photon extraction (luminescence), remain to be addressed for nanopixel applications [47]. Once this LSPR is excited, the strong optical near-field is enhanced around the nanoparticle owing to the collective and coherent oscillation and excitation of free electrons of the metal nanoparticle [17, 36, 51]. Thus, the incident photons are absorbed and converted to heat (phonons) [52], [53], [54], and at the same time, the photons are strongly scattered, resulting in stable colors. The extinction *E* is simply expressed by [55, 56]

(1) $$E\propto \frac{\epsilon_{i}}{{\left(\epsilon_{r}+\chi n^{2}\right)}^{2}+\epsilon_{i}^{2}},$$

where $\epsilon_{r}$ , $\epsilon_{i}$ , and χ are, respectively, the real and imaginary terms of the permittivity and a geometrical factor of the metal nanoparticle, n is the refractive index of the medium surrounding the nanoparticle. The maximal E refers to the LSPR that occurs when

(2) $$\epsilon_{r}\left(\lambda^{*}\right)=-\chi n^{2}.$$

This shows that the LSPR wavelength $\lambda^{*}$ is a function of the metal nanoparticle’s shape in terms of χ , the metal nanoparticle’s material composition in terms of $\epsilon_{r}$ , and the refractive index of the surrounding medium n . Advances in nanofabrication offer nanostructures with considerable flexibility in engineering all these parameters and thus continuously extend the possible LSPR range across the whole visible regime. For instance, gold and silver structures are grown with various shapes ranging from simple spheres to complex rods, stars, and helices [57], [58], [59], [60], [61], [62], [63], [64], [65], [66], [67], [68], [69], [70] and/or arranged in an ensemble [34, 42, 43, 71] or array [44], [45], [46] to show plasmonic colors from the near-ultraviolet to near-infrared. Aluminum [72], gallium [73], and magnesium [74] have emerged as ultraviolet plasmonic materials [75, 76]. More crucially, if one or more of the three parameters (i.e., χ , $\epsilon_{r}$ , n ) is reversely tunable, such as in conjunction with responsive materials, the static resonant color set by these parameters becomes active (top panels in Figure 2B–D). Here, we summarize the recent achievements of such color-changing plasmonic nanopixels. Although Eq. (2) does not fully describe all possible complex geometries and optical interactions, it is nevertheless a helpful guide. The equation allows plasmonic nanopixels to be categorized and we thus use it in discussing specific examples where (i) Δχ , relating to the shape (Section 3.2), (ii) $\Delta \epsilon_{r}$ , relating to the dielectric function of the nanoparticle (Section 3.3), or (iii) Δn , relating to optical properties of the medium surrounding the nanoparticle (Section 3.1), is tunable in response to an external stimulus.

### 2.2 Dielectric nanoscatterers

Nanoscale scatterers can generally be described on the basis of Rayleigh or Mie scattering [83–85]. The Rayleigh scattering approximation explains elastic scattering of light with scatterers that are much smaller than the wavelength of the incident light (i.e., a particle size less than 1/10 of the wavelength) [84, 86], [87], [88], [89]. Meanwhile, for larger particles whose size is within orders of magnitude of the wavelength, the Mie scattering approximation gives exact solutions for absorption and scattering by the nanoparticle (bottom panel in Figure 2A), even in the case that the material, size, and refractive index of the medium are changed [19, 80, 82, 84]. By solving the Maxwell equation with the boundary conditions at the particle surface, the scattering and extinction cross-sections of the nanoparticle dispersed in a linear, isotropic, and homogeneous medium (with refractive index n ) are expressed as [87, 90]

(3) $$C_{\text{sca}}=\frac{2\pi }{k^{2}}\overset{\infty }{\underset{m=1}{\sum }}\left(2m+1\right)\left({\left|a_{m}\right|}^{2}+{\left|b_{m}\right|}^{2}\right),$$

(4) $$C_{\text{ext}}=\frac{2\pi }{k^{2}}\overset{\infty }{\underset{m=1}{\sum }}\left(2m+1\right)\text{Re}\left\{a_{m}+b_{m}\right\},$$

where k is the wavenumber and $a_{m}$ and $b_{m}$ are the electric and magnetic Mie scattering coefficients that are also functions of *n*, the refractive index surrounding the nanoparticle. Additionally, the absorption cross-section can be obtained by subtracting the scattering cross-section from the extinction cross-section (i.e., $C_{ext}=C_{abs}+C_{sca}$ ). These equations show that the Mie resonance is a function of the scattering coefficient as well as n , the refractive index of the medium surrounding the nanoparticle.

Among diverse dielectric nanoparticles, including TiO_2_ [82, 91], VO_2_ [80], Ge [92], [93], [94], Te [92], and silicon nanostructures are representative Mie scatterers that exhibit intriguing optical interactions of magnetic and electric resonances in the visible-light regime owing to the characteristic of the displacement currents formed in the body of the nanoparticles [19, 87, 90, 95], [96], [97], [98], [99], [100]. From the Mie solution, the magnetic-dipole resonance of a silicon sphere occurs at an approximate wavelength of $\lambda =2n_{d}r$ where λ is the wavelength of resonance, $n_{d}$ is the refractive index of the nanosphere, and r is the radius of the dielectric nanosphere [90]. Once $n_{d}$ of the material is fixed, there is a resonance condition of the dielectric nanosphere as a function of the nanosphere size in the visible (bottom panel in Figure 2B). Furthermore, nanoscatterers with larger dimensions excite higher-order modes, such as electric- and magnetic-quadrupole resonances [19, 95, 96, 101, 102]. As a result, the Mie resonant coloration is mainly determined by three parameters, namely the shape (or size r , i.e., χ in plasmonic resonance) and material composition $n_{d}$ , (proportional to $\epsilon_{r}$ ) of the nanoparticle, and n of the surrounding optical environment. Similar to the case of plasmonic resonance, Mie scatterers can be activated by making one of these parameters respond to the external stimulus, as summarized in Section 4.1 (bottom panels in Figure 2B–D). For simplicity, we here use Δχ , $\Delta \epsilon_{r}$ , and Δn for both plasmonic and Mie resonances.

## 3 Responsive plasmonic nanopixels

The plasmonic nanoscatterers act as nanopixels that modulate the color intensity (i.e., on/off switching) or wavelength (i.e., dynamics) in conjunction with responsive materials, changing their optical property in response to the associated type of external stimuli. This section presents modulations according to the refractive index of the medium surrounding the plasmonic nanoscatterers ( Δn , Section 3.1) and the shape ( Δχ , Section 3.2) and refractive index ( $\Delta \epsilon_{r}$ , Section 3.3) of the plasmonic nanoscatterers, as the three critical parameters for active coloration.

### 3.1 Modulation on the optical environment surrounding nanoscatterers

Once fabricated, the nanoparticles and structures do not readily change shape or material composition, and the plasmonic nanopixels are thus developed intensively with functional materials to modulate the refractive index of their surrounds (∆*n*) reversibly [103], [104], [105], [106], [107], [108], [109], [110], [111], [112], [113], [114], [115], [116], [117], [118], [119], [120]. If the surrounds are filled with responsive materials, the plasmonic resonance becomes active. The most effective approach is formulating a shell layer around the nanoparticle or filling a gap in the plasmonic ensemble or multilayered structures (e.g., the nanoparticle-on-mirror construct) with responsive materials. Depending on the type of responsive material, various stimuli can be used to tune the scattered colors. Here, we highlight recent developments of responsive plasmonic nanopixels with electrochromic materials (Section 3.1.1) and other responsive materials (Section 3.1.2).

#### 3.1.1 Electrochromic plasmonic nanopixels

**Figure 3:**
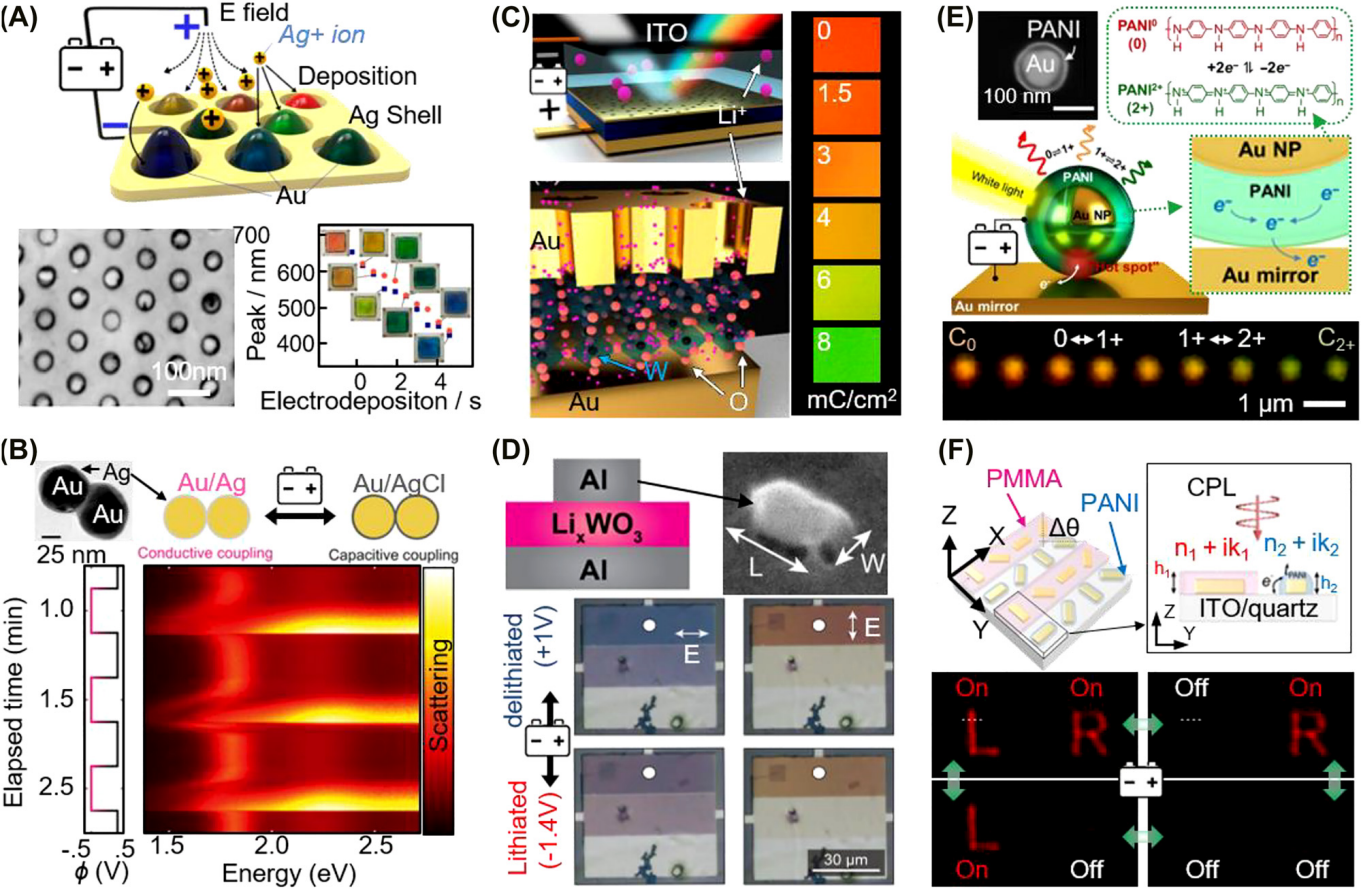
Electrochromic plasmonic nanopixels with modulation of the refractive index Δ*n* of the surrounds of the nanoparticle. Color dynamics achieved by (A) depositing/etching Ag on Au nanodomes (reproduced with permission [112], Copyright 2016, American Chemical Society), (B) changing material state from Ag (metal) to AgCl (dielectric) in the gap between two plasmonic Au nanoparticles (reproduced with permission [81], Copyright 2015, American Association for the Advancement of Science, (C–D) doping lithium ion into the WO_3_ gap of the plasmonic cavity (reproduced with permission [109, 111], Copyright 2019, American Chemical Society and 2020, American Chemical Society), and (E–F) redox reaction of the polyaniline in the plasmonic system (reproduced with permission [108, 110], Copyright 2021, American Association for the Advancement of Science and 2019, American Association for the Advancement of Science).

Electrochemical means have been used to make plasmonic nanoparticles function as small optical switches/pixels (Figure 3). The first examples are of Ag metal, where Au nanostructures coated with Ag shells show wide color dynamics through electrochemical control of the Ag shell thickness (Figure 3A) [112] or redox state (Figure 3B) [81]. The former approach uses an array of 50 nm Au nanodomes coated with a gel electrolyte containing Ag^+^ ions, which are deposited or etched along the surface of the Au nanodomes according to the external voltage applied (−1 to 1 V) [112]. The thickness of the Ag layer can be gradually controlled within the range of 0–40 nm to change the color between red and blue. Figure 3B shows the plasmonic Au dimer encapsulated in the Ag shell layer, which varies via the redox chemistry between Ag metal and AgCl dielectric states (−5 to 5 V) [81]. As a result, dimers can switch between conductive and capacitive resonance, leading to a color change from red (742 nm) to orange (620 nm). However, when Ag is repeatedly stripped/redeposited or oxidized/reduced, the ionic diffusion is slow and leads to rapid nanoscale morphological changes, and these approaches thus suffer from poor long-term reproducibility (within 1 month) and slow switching (with a period longer than 0.5 s).

Another approach is to combine plasmonic nanoparticles with metal oxides (Figure 3C and D) [109, 111]. Metal oxides, such as MoO_3_, V_2_O_5_, and WO_3_, change their refractive indices as a function of ion doping into their bulk matrix, and they are therefore used to fill gaps in plasmonic structures as an active medium with controllable Δn . For instance, a 120 nm WO_3_ layer, doped with Li-ion (Li^+^), can be used as a gap between 20 nm Au nanoholes and an Au mirror by applying a voltage in the range of −1.4 to 1 V (Figure 3C) [111]. Such doping (i.e., lithiation) changes the phase of the WO_3_ (insulating state) to LiWO_3_ (metallic state) and changes the associated complex refractive indices, *e.g.* n from 1.94 to 1.66 and k from 0 to 0.5 at λ = 632 nm. Therefore, as the degree of lithiation increases, the plasmonic color changes from red to green (595–505 nm). The nanopixels in Figure 3D work by the same principle to change the plasmonic colors supported by the rectangular Al nanoparticles on an Al mirror having a 17 nm-thick WO_3_ gap [109]. The voltage range of −1.4 to +1 V is applied to modulate the doping level of Li+, and the associated *n* is thereby modulated between 2.1 and 1.9. Such ∆*n* modulation changes the colors with a dynamic range of 58 nm in the visible. The Al rectangular particles intrinsically have polarization-dependent resonance along their structural long and short axes, and they therefore provide another degree of freedom with which to modulate coloration (see Section 3.2 for details).

The color change of plasmonic nanoparticles through electrochemistry can be dramatic when conductive polymers are combined (Figure 3E–F) [108, 110]. For instance, polypyrrole [121, 122], poly(3,4-ethylene dioxythiophene) (PEDOT) [121, 123, 124], and polyaniline (PANI) [108, 110, 121, 125] are typically integrated with diverse plasmonic core–shell nanoparticles [121, 126], [127], [128], [129] and multilayered coupling systems [110, 130], [131], [132], [133] and used to develop electrochromic devices. In particular, PANI has the most considerable change in the refractive index $\left(\Delta n_{max}=\text{ca. }0.6\right)$ in the visible upon a redox reaction (often involving a voltage sweep from −0.2 to 0.6 V). For instance, Au nanoparticles coated with PANI as a shell layer are coated on Au film, where the PANI acts as an active gap medium (Figure 3E) [110]. Such electrochromic nanoparticle-on-mirror structures are fabricated through scalable means (e.g., conventional aerosol printing) from the single-nanoparticle level to multi-centimeter-scale films, even on flexible plastics [125]. Thanks to the large of Δn the PANI, this leads to an appreciable change in the colors supported by such plasmonic nanopixels from red to green (i.e., a dynamic wavelength larger than 70 nm in the visible regime) at the single nanoparticle level (having a spatial dimension less than 100 nm). These color-changing nanopixels require a voltage less than 1 V for full-color modulation with a near video refresh rate. PANI intrinsically possesses a bistable characteristic and thus retains plasmonic color with no external voltage (i.e., it has a long retention time). Low-power-driven electrochromic devices can thus be realized, but the dynamic color should be extended to blue for commercial display applications. Interestingly, the same mechanism can be applied to activate holographic metasurfaces (Figure 3F) [108]. The array of meta-atoms is patterned and coated with PANI onto an even number of sets of the meta-atoms (being the active part). The remaining meta-atoms are patterned orthogonally against the active part and covered with polymethyl methacrylate (PMMA, having constant *n* = 1.5), acting as a passive part. The modulation of the complex refractive indices of the PANI coated on the meta-atoms in the active part allows the control of the phase (i.e., polarization) state of the light traveling through the meta-atoms, eventually resulting in modulation in the holographic images (see Section 5.3 for the application of active holograms).

#### 3.1.2 Other responsive plasmonic nanopixels

**Figure 4:**
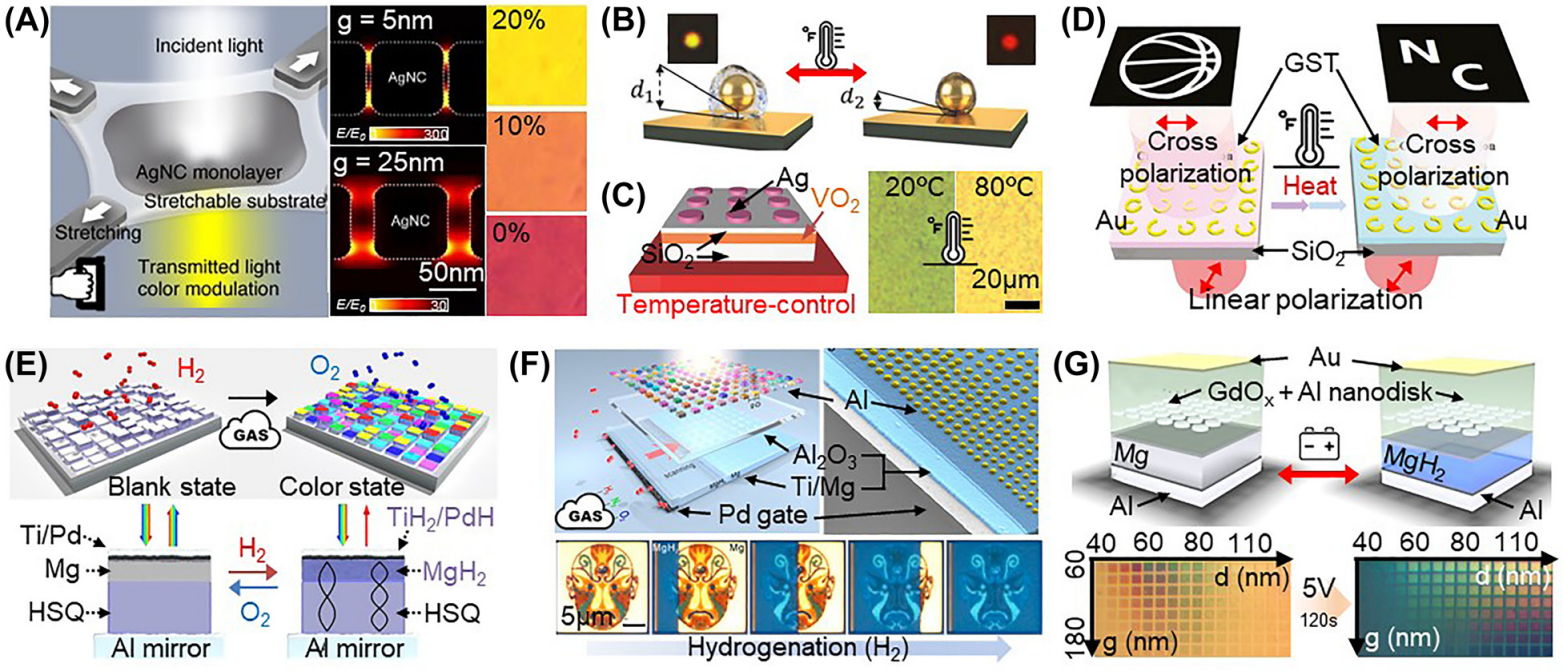
Various responsive plasmonic nanopixels with the refractive index modulation, Δn surrounding the nanoparticle. Mechanochromic nanopixels driven by (A) physical strain (reproduced with permission [107], Copyright 2018, Wiley-VCH) and (B) heat (or laser) (reproduced with permission [106], Copyright 2018, Wiley-VCH). Thermo-chromic nanopixels for (C) coloration (reproduced with permission [105], Copyright 2018, Wiley-VCH), (D) dynamic holographic images (reproduced with permission [120], Copyright 2020, Walter de Gruyter GmbH), and (E–G) hydrogenation-based color dynamics (reproduced with permission [117], [118], [119], Copyright, 2019, Springer Nature, 2018 American Chemical Society, and 2017, American Chemical Society).

Other external stimuli can be used to drive plasmonic coloration, and we therefore highlight recent responsive plasmonic nanopixels with mechanical [107] or thermal forces [105, 106, 120] or gas adsorption [117], [118], [119] (Figure 4). When two or more plasmonic systems (either between nanoparticles or as part of a nanoparticle-on-mirror construct) are positioned with a tiny gap (with the particle size being less than 0.1), a strong optical field coupling is generated in the gap, forming what is known as a hot spot [110]. This hot spot leads to a strong additional coupled resonance [110, 134, 135], which not only becomes the dominant scattered color but is also sensitive to changes in the spatial dimension and refractive index of the gap [110, 135, 136].

The plasmonic color can thus be modulated by a tiny change in the gap distance in conjunction with responsive polymers [107]. By dispersing the plasmonic Ag nanocubes in a stretchable polymer matrix (e.g., polydimethylsiloxane, PDMS), bi-axially stretching the polymer (by 20%) via an external mechanical force has been shown to change the spatial dimension of the gap from 5.8 to 24.8 nm (i.e., there is larger inter-particle spacing) with a negligible change in the effective refractive index in the PDMS matrix between the Ag nanocubes (Figure 4A). As a result, these nanopixels exhibit color dynamics from magenta to yellow. This gap-controlled tuning mechanism indeed works with heat (or light) using thermo-responsive polymers, such as poly(2-(2-methoxyethoxy)ethyl methacrylate) [137], poly(DEGA-co-OEGA) [138], and poly(N-isopropyl acrylamide) (PNIPAM) [106, 139]. These polymers contract or expand in response to heat and are thus used to fill the gap of the plasmonic structures in developing thermo-responsive plasmonic nanopixels. For instance, the PNIPAM-coated Au nanoparticles-on-mirror construct in Figure 4B has a change in the gap distance due to heating (or laser illumination, and thus eventually heating, for photothermal interaction). When the temperature exceeds 32 °C (i.e., the lower critical solution temperature) [106], the PNIPAM is contracted, and this shifts the top Au nanoparticles close to the mirror side (i.e., there is a decrease in the gap from 20 nm at 25 °C to 8 nm at 40 °C, thus changing the color from 590 to 645 nm. Note that, according to effective medium theory [85, 106], the effective refractive index of the PNIPAM changes from *n*_25 °C_ = 1.34 to *n*_40 °C_ = 1.42 due to the change in the water density in the polymer gap [106], but the numerical simulation predicts negligible color dynamics (a shift of approximately 15 nm) [106]. Therefore, the color modulation here dominantly relies on the physical change in the plasmonic gaps.

A color change via modulation of the refractive index is also possible with thermally responsive phase transition materials, such as vanadium dioxide (VO_2_) [113, 140], [141], [142] and Ge_2_Sb_2_Te_5_ (GST) [120]. These materials undergo a phase transition from insulating to metallic as a function of the heat, typically in the range of 20–80 °C, resulting in the modulation of the complex refractive index [105, 120]. Thermally driven plasmonic nanopixels can therefore be realized when such material is used as a gap between plasmonic structures (Figure 4C and D). For instance, an array of 120 nm Ag nanodisks with 300 nm spacing have been patterned on SiO_2_/VO_2_ multilayers (Figure 4C), where VO_2_ acts as a thermo-responsive medium that changes the refractive index from 3.09 to 2.63 when the sample is heated from 27 to 82 °C [105]. This dynamic modulates the color from 560 to 540 nm. Another example is split-ring resonators of various sizes (i.e., a radius of 180–370 nm and a fixed width of 80 nm) patterned on GST (Figure 4D) [120]. GST changes the complex refractive index (*n* = 3.707–7.37, *k* = 0.0105–0.67) via heating (above 160 °C) and modulates the phase and amplitude of the incident light passing through it. Therefore, completely different images with little crosstalk are projected with the same split-ring resonators. However, most thermally responsive phase-change materials have larger Δn in the near-infrared than in the visible, such that they are often inappropriate for plasmonic color dynamics in the visible, especially in the short-wavelength regime (e.g., the blue regime).

An alternative approach is to use phase-change materials that interact with a gas, such as hydrogen (Figure 4E–G). Magnesium, for example, is used as a gap between Al nanoparticles and an Al mirror (Figure 4E) [119] or as a bottom mirror for plasmonic coupling (Figure 4F) [118]. The Mg works as a metallic mirror and thus blocks the upcoming light from striking the resonators underneath, reflecting all light rays. However, if hydrogen (20%) is applied to the Mg for more than 10 h, Mg changes to transparent (dielectric) MgH_2_, through which light can pass. Hence, the colorful image plane set by the array of the cavity resonators appears (Figure 4E) [119]. Meanwhile, the Mg layer at the bottom acts as a plasmonic metallic mirror to resonate the light within the cavity with the nanoparticles on top, and the interaction provides vivid colors (Figure 4F) [118]. Then, through the hydrogenation of the Mg, the dielectric MgH_2_ forms and breaks the plasmonic coupling, erasing all colorations. Such hydrogen-gas-driven active plasmonic coloration can be used for colorimetric sensing and display applications. Still, the major bottleneck for industrial application is the gas-based operation and slow switching time. A recently proposed means by which to overcome these problems is to directly integrate a solid-state proton source into plasmonic devices (Figure 4G) [117]. An array of Al nanodisks is embedded in gadolinium oxide (GdO_*x*_) and sandwiched with Au (top) and Mg/Al (bottom) layers. When a voltage of +5 V is applied for 10 ms, the nearby moisture at the interface between GdO_*x*_ and Au is continuously decomposed into oxygen molecules (O_2_) and hydrogen ions (H^+^). These protons then flow through the GdO_*x*_ and reach the Mg mirror, converting metallic Mg into transparent MgH_*x*_. This allows the external light to reach the lower Al film and resonate inside the cavity, leading to vivid colors. Restoration is realized by applying a voltage of −2 V. The Mg-based plasmonic nanopixels have vivid and rich color performance with high resolution and fast switching. However, several problems, such as low throughput and switching repeatability, need to be overcome for industrial applications.

### 3.2 Modulation on the shape of nanoscatterers

The modulation of shape factor Δχ of the metal nanoparticles is another branch of active plasmonic coloration. Δχ can be achieved with polarization-dependent light–matter interaction of the anisotropic nanoparticles, e.g., nanorods, prisms, and helices, whose structural long and short axes show different colors due to the difference in their χ . In contrast, the change in the physical shape of the nanoparticles is also another means to modulate Δχ , so here these two approaches are mainly summarized.

**Figure 5:**
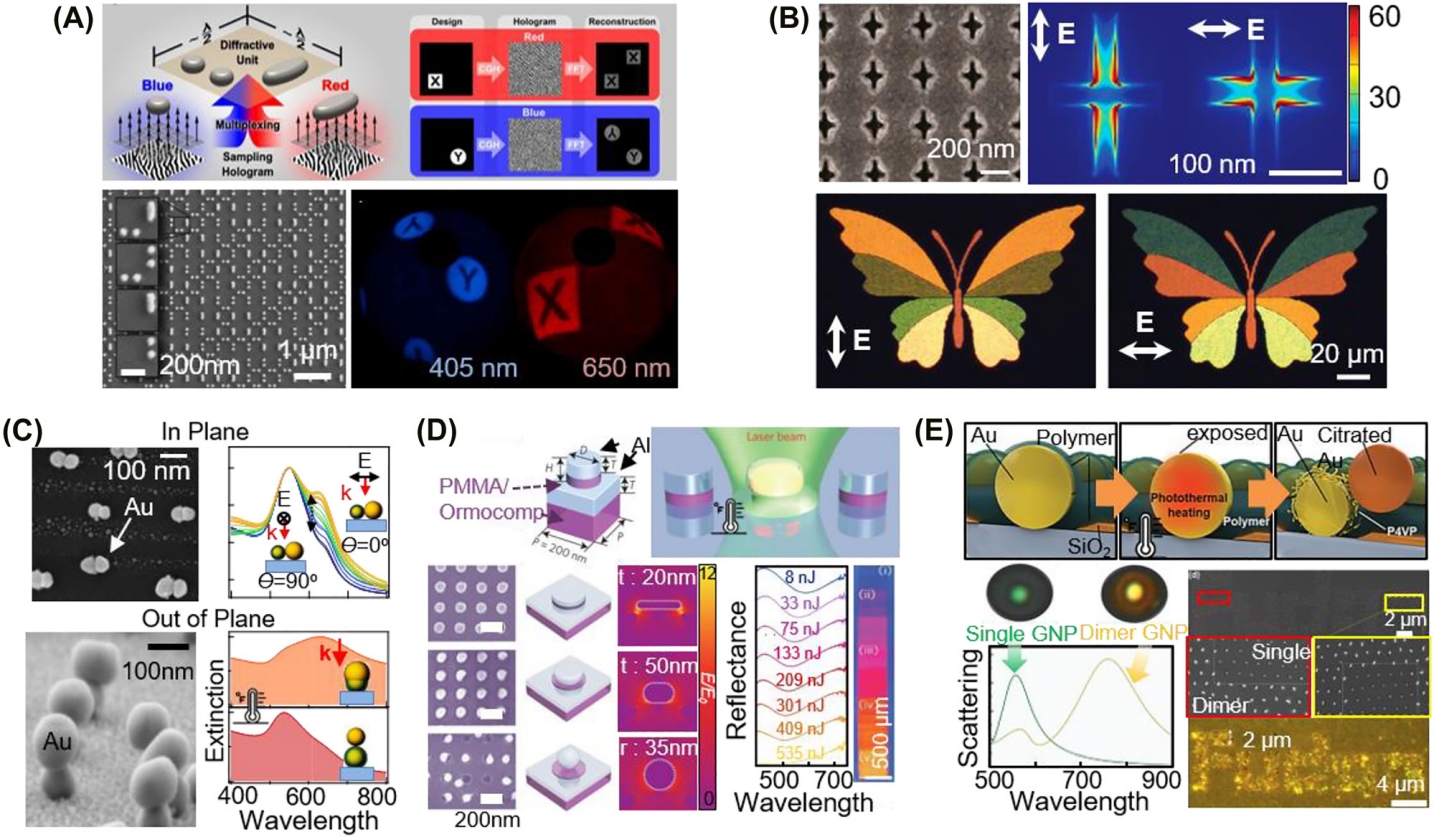
Responsive plasmonic nanopixels with modulation of the shape factor, Δχ , of the nanoparticle: polarization-dependent coloration with (A) Ag nanostructures and (B) Al nanoholes (reproduced with permission [77, 143], Copyright 2014, Europe PMC and 2016, American Chemical Society), thermally driven shape modulation for coloration with (C) plasmonic dimers (reproduced with permission [144], Copyright 2019, American Chemical Society), (D) Al nanoparticles on Al mirror with PMMA/Ormocomp polymer spacers (reproduced with permission [145], Copyright 2016, American Chemical Society), and (E) gluing of nanoparticles with heat (reproduced with permission [146], Copyright 2021, Wiley-VCH).

An increase in length or shape complexity along one or more axes of nanoparticles having the forms of rods [57, 58, 63, 71], prisms [58, 63, 71, 147], stars [58], and helices [58, 63, 148, 149], introduces additional LSPR modes along the long axis; these are called longitudinal modes [57, 58]. Typically, as the anisotropic geometry increases the polarizability along the long axis of the plasmonic nanoparticle, it red-shifts the resonance color with respect to that of the transverse mode (LSPR along the short axis) [57, 58]. Thus, a simple change in the linear polarization of the light along the axis of the anisotropic nanoparticles can actively modulate the plasmonic color. For instance, various combinations of anisotropic Ag nanostructures have been patterned on Si wafer to project polarization-dependent holographic three-dimensional (3D) images (Figure 5A) [77]. Furthermore, the nanoparticles reveal the scattered colors with no interference between them, and the wavelength-dependent coloration can thus be readily achieved by matching the longitudinal LSPR modes of the nanoparticles with target colors (here, blue at 405 nm and red at 650 nm). There are many different types of plasmonic coloration based on this concept [150], [151], [152], but we here present another representative case, an Al film with hole cavities (negative pattern, Figure 5B) [143]. By dynamically controlling the external light source, plasmonic color modulation is possible through the modulation of the shape factor Δχ , enabling the generation of two or more colors supported by the individual single nanopixels. The nanohole cavities pattered on Al film in Figure 5B have different vertical (203 ± 3 nm) and horizontal (120 ± 5 nm) lengths, and various colors in the visible ranging from 485 to 610 nm can thus be expressed with predefined asymmetrical nanopixels by modulating the angle and polarization of the incident light without the physical modulation of the nanoparticle shape.

Another route by which to address Δχ is the deformation of the physical shape of the plasmonic nanoparticles. For instance, Au nanoparticles covered with Au nanocaps (separated with thin alumina) can deform in shape as near-touching Au dimers via heating at 300 °C (Figure 5C) [144]. The initial elongated fused nanoparticles show LSPR at 633 nm, similar to LSPR for a large nanosphere, but this LSPR changes to 536 nm once dimers are formed as they offer coupled LSPR colors, enabling polarization-dependent LSPR switching (top panel in Figure 5C). The shape deformation of the plasmonic nanostructures can also be possible with laser heating (8–535 nJ at 532 nm) (Figure 5D) [145]. Al nanodisks are patterned on the Al mirror separated with polymer pillars, and they therefore show initially coupled resonant colors at 532 nm. When the laser is illuminated, the nanodisk and polymer pillar melt together and deform in shape into a sphere, affecting the LSPR coupled-mode and thus changing the color, such as from blue to yellow. Meanwhile, a laser (1.0 mW at 532 nm) has been used instead to glue two nanoparticles together in effectively modulating χ ; i.e., a transition from a single nanoparticle to a dimer (Figure 5E) [146]. Thermally deformable polymers (polystyrene) have been used to cover the surface of Au nanoparticles (diameter of 80 nm) arranged with a regular lattice (spacing of 500 nm). A laser then partially peels away the polymers to trap other second nanoparticles electrostatically with the first nanoparticles. While the single nanoparticles alone show color at 565 nm, the dimers give rise to a red-shifted distinct color at 760 nm. However, active coloration is irreversible using such heating-based shape deformation or gluing the nanoparticles, and the initial colors cannot be recovered unless a reversible approach is developed.

### 3.3 Modulation on the material state of nanoscatterers

**Figure 6:**
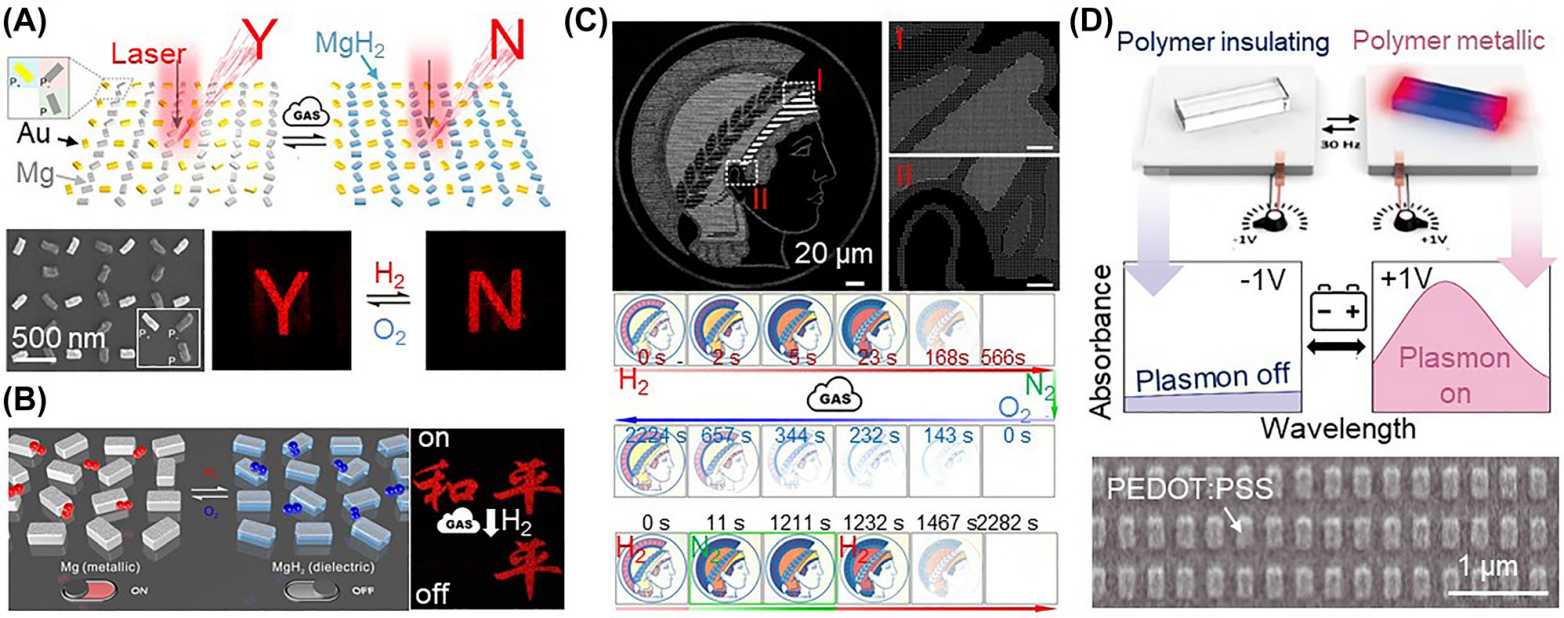
Responsive plasmonic nanopixels with the modulation of the real part of the dielectric constant $\Delta \epsilon_{r}$ of the nanoparticle: hydrogenation-driven phase change in the Mg nanostructures for (A–B) image change and on/off switching (produced with permission [78, 154], Copyright 2018, American Association for the Advancement of Science and 2018, American Chemical Society), and (C) color bleaching dynamics by gas reaction (reproduced with permission [155], Copyright 2017, Springer Nature). (D) electrochromic polymer nanopixels for plasmonic color dynamics by changing the redox states of the conductive polymer (reproduced with permission [153], Copyright 2021, American Association for the Advancement of Science).

The third parameter for active plasmonic nanopixels is $\Delta \epsilon_{r}$ , the change in the real part of the dielectric constant of the plasmonic nanoparticle, which is a material property. Suppose that the dielectric constant of the plasmonic material can be modulated between several dielectric states actively and reversibly. In this case, the plasmonic nanopixels are functional for on/off switching [78, 153, 154] and tunable coloration [155]. Phase-change materials and responsive polymers are potential candidates, but they are often plasmonically inactive, except for (but not limited to) some cases discussed in this review. We highlight recent progress in developing active coloration based on the material phase transition of plasmonic nanostructures (Figure 6).

As seen in Section 3.1.2, Mg is a metal that transforms into dielectric MgH_2_ in response to hydrogen and can reversely return to the metal state by interacting with oxygen [78, 117, 119, 154, 155]. More importantly, Mg nanoparticles can give rise to strong plasmonic features, typically in the ultraviolet [74], such that they can be directly used as phase-change plasmonic nanopixels. For example, a pair of Au and Mg nanorods with an orthogonal arrangement is patterned as the metasurface, where each pair works as an individual nanopixel for color dynamics (Figure 6A) [154]. Depending on the type of gas injected (either hydrogen or oxygen), the plasmonic response of individual Mg nanorods can reversibly switch between on and off states whereas there is no change in those of the Au nanorods. Thus, on the basis of this on/off switching with pixelized coloration, the metasurface can project two completely different holographic image planes. Furthermore, the gradual phase transition is possible due to the partial capping Cr layer surrounding the Mg nanopixels (Figure 6B,C) [78, 155]. The partial capping layer on the Mg nanopixels prevents an instant reaction throughout the materials and thus slows down the transition time from 1 min to 10 min. An intermediate transition between Mg and MgH_2_ is then possible, and the stepwise dielectric constant modulation in plasmonic materials thus enables multiple steps in color transitions (Figure 6B) [78]. In addition to $\Delta \epsilon_{r}$ , the Mg nanoparticles expand and shrink (by a maximum of 32%) during the gas reaction, Δχ , which contributes to tuning the coloration although the dynamic remains slow (taking longer than several seconds) (Figure 6C) [155].

Meanwhile, a new concept of an electrochromic plasmonic with conductive polymers has recently emerged (Figure 6D) [153]. One of the most well-known conductive polymers, PEDOT:PSS, intrinsically possesses metallic characteristics in the infrared, but surprisingly, this has never been reported according to Ref [153]. Such nanostructures are plasmonically active. By applying a voltage of +1 V, PEDOT:PSS nanorods (width: 160 nm, length: 300 nm, height: 90 nm) can be fully oxidized to be metallic, having plasmonic resonance at approximately *λ* = 2.2 μm. Conversely, they can be fully reduced with a reverse bias of −1 V, becoming a dielectric for which there is no resonance. This opens up a new research direction in investigating the plasmonic behavior of conductive (and possibly other) polymers. In particular, generating plasmonic resonance in the visible with polymer nanostructures might be an appreciable challenge.

## 4 Other responsive nanopixels

Nanopixels showing Mie resonance recently found a use for active coloration and holographic metasurfaces due to their strong material stability and deficient absorption in the visible regime [19, 156], [157], [158]. However, because these nanopixels also have modulation parameters relating to plasmonic resonance (i.e., parameters of their material composition and particle shape and the refractive index of the surrounds), there are many similarities in the active mechanisms in many cases [82, 91, 159], [160], [161], and we here avoid similar mechanisms and focus on recent developments with concepts not covered in the previous sections (Section 4.1). Furthermore, we summarize emerging hybrid plasmonic (or Mie) resonance nanopixels in conjunction with light conversion bandgap nanoparticles; i.e., down- or up-conversion nanoparticles (Section 4.2).

### 4.1 Mie resonant nanopixels

**Figure 7:**
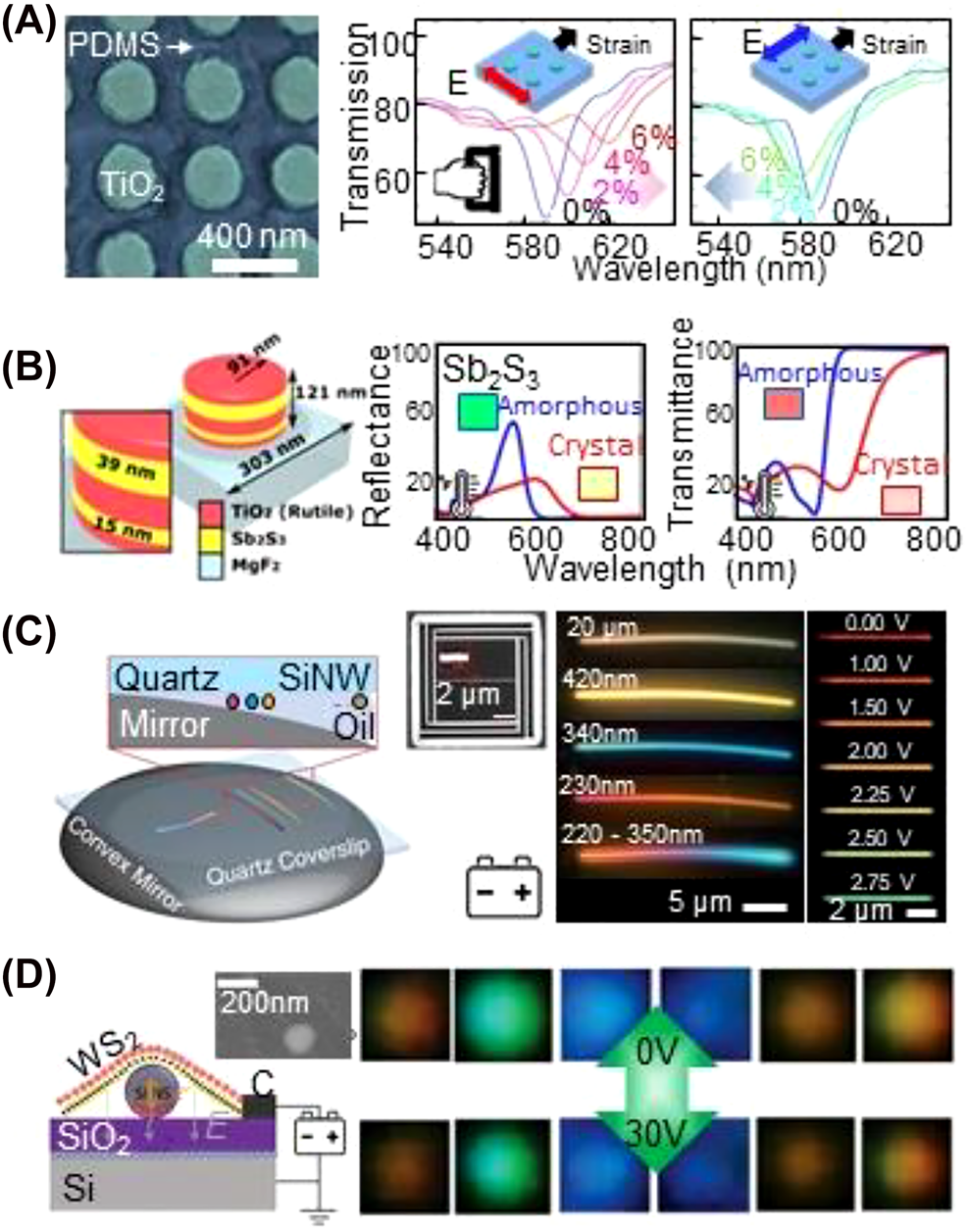
Various responsive Mie resonance nanopixels with modulation of the refractive index Δn of the surrounds of the nanoparticle: (A) mechano-chromic nanopixels driven by physical strain (reproduced with permission [91], copyright 2016, American Chemical Society), (B) thermo-chromic nanopixels for dichroic coloration (reproduced with permission [82], Copyright 2020, Optica Publishing Group), (C–D) electrochromic nanopixels obtained by (C) controlling the gap distance between the dielectric rod scatterer and the mirror underneath (reproduced with permission [159], Copyright 2017, American Association for the Advancement of Science), and (D) sagging the WS_2_ layer to wrap the Mie scatterer (reproduced with permission [160], Copyright 2021, Wiley-VCH).

Among the three modulation strategies for plasmonic nanopixels, a rational approach to reversibly modulating the Mie scattering color with large flexibility of the type of stimuli is to use Δn , the change in the refractive index of the surrounds of the nanoscatterers. We here present four representative cases of Δn for mechanochromic [91], thermochromic [82], and electrochromic systems [159, 160] with Mie resonances (Figure 7). The first case is that of the mechanochromic device (Figure 7A) [91]. The reversible modulation of a distance (displacement of 0–0.6 μm) between TiO_2_ dielectric nanoscatterers (having a diameter of 190 nm) embedded in a polymer matrix (here PDMS) changes the associated scattered color from 591 to 620 nm. If strain is applied along only one axis, then the lattice symmetry of the scatterers is broken and the color becomes polarization dependent. For instance, light linearly polarized orthogonal to the axis of the strain applied for 6% extension in length increases the transmittance from 47% to 78% at *λ* = 591 nm, while maintaining the transmittance of the linearly polarized light parallel to the applied strain axis (approximately 29%). The second case is active Mie resonant color modulation using a thermoresponsive phase-transition material (Figure 7B) [82]. Here, 300 nm TiO_2_ dielectric nanocylinders (having a total thickness of 67 nm) are alternately stacked with 39- and 15 nm-thick Sb_2_S_3_ layers, and their refractive indices depend on the crystal phase. The amorphous Sb_2_S_3_ (*n* = 3.58) has a refractive index similar to that of TiO_2_ (rutile, *n* = 3.02) in most of the visible regime, while resonantly reflecting light with a wavelength *λ* = 520 nm by approximately 60% through Mie resonance of the electric dipole. Meanwhile, when heating the device at 630 °C for 2.7 ns, the Sb_2_S_3_ layer becomes crystallized such that the effective refractive index changes to *n* = 4.35 with a large increase in k (from 0.3 to 1.1), resulting in a redshift to 580 nm. However, several issues, such as the high operation temperature and thus short lifetime, need to be addressed for such heating-based modulations [162], [163], [164].

Mie resonance between two or more dielectric scatterers accompanies resonance coupling, and this can be tunable, analogous to modulation in gap plasmonics (i.e., a change in the physical dimension and/or refractive index of the gap) [82, 159, 165]. For example, Si nanowires (50 nm in width and 25 μm in length) have been deposited on a flat quartz substrate, then flipped and suspended on an oil phase towards a bottom convex Al mirror having a large radius of curvature (10 mm) (left panel in Figure 7C) [159]. If the top quartz substrate laterally slides towards the middle side of the Al mirror, reducing the distance between the mirror and Si nanowires from 20 mm to 230 nm, the coupling of the scattered lights sequentially become white (broadband resonance), yellow (590 nm), blue (490 nm), and red (670 nm). This is due to the change in the interference between the light reflected from the mirror and the scattered light supported by the nanowire whose color is deliberately set by the electric dipole resonance owing to its small width (50 nm). Such a modulation concept has also been demonstrated for reversible electrical switching, by integrating a Si nanowire with an electromechanical actuator suspended on the Si mirror substrate (right panel in Figure 7C). When an applied voltage gradually increases from 0 to 2.75 V, the suspended Si nanowire is steadily pulled down to the bottom mirror (i.e., there is a change in the gap distance of 150 nm), leading to a color change from 700 to 520 nm. This electrochromic function with Mie scattering has excellent potential for integrated optoelectronic devices, but the overall size for coloration is on the micron scale (far bigger than that for plasmonic scattering).

Other intriguing electrochromic nanopixels using Mie resonance are Si nanoparticles sandwiched between a WS_2_ monolayer (top electrode) and SiO_2_ (200–300 nm)/Si substrate (bottom electrode) (Figure 7D) [160]. The application of an electric voltage (30 V) to the device draws the top WS_2_ layer down towards the bottom electrode to wrap around the Si nanoparticles. This large sagging of the WS_2_ monolayer (42–177 nm) due to the increase in capacitance inside the Si nanoparticle sharply weakens the enhanced electric and magnetic fields of the Mie resonance surrounding the nanoparticle and thus suppresses far-field scattering colors. For example, 168- and 140 nm Si nanoparticles respectively show magnetic-dipole scattering at 652 and 586 nm, which attenuates by up to 40% upon applying a voltage. Each nanoparticle thus performs as an on/off optical switch, which is potentially useful for display applications. However, several significant challenges, such as an extremely high voltage operation and low fabrication yields, remain.

### 4.2 Hybrid nanopixels for light conversion

**Figure 8:**
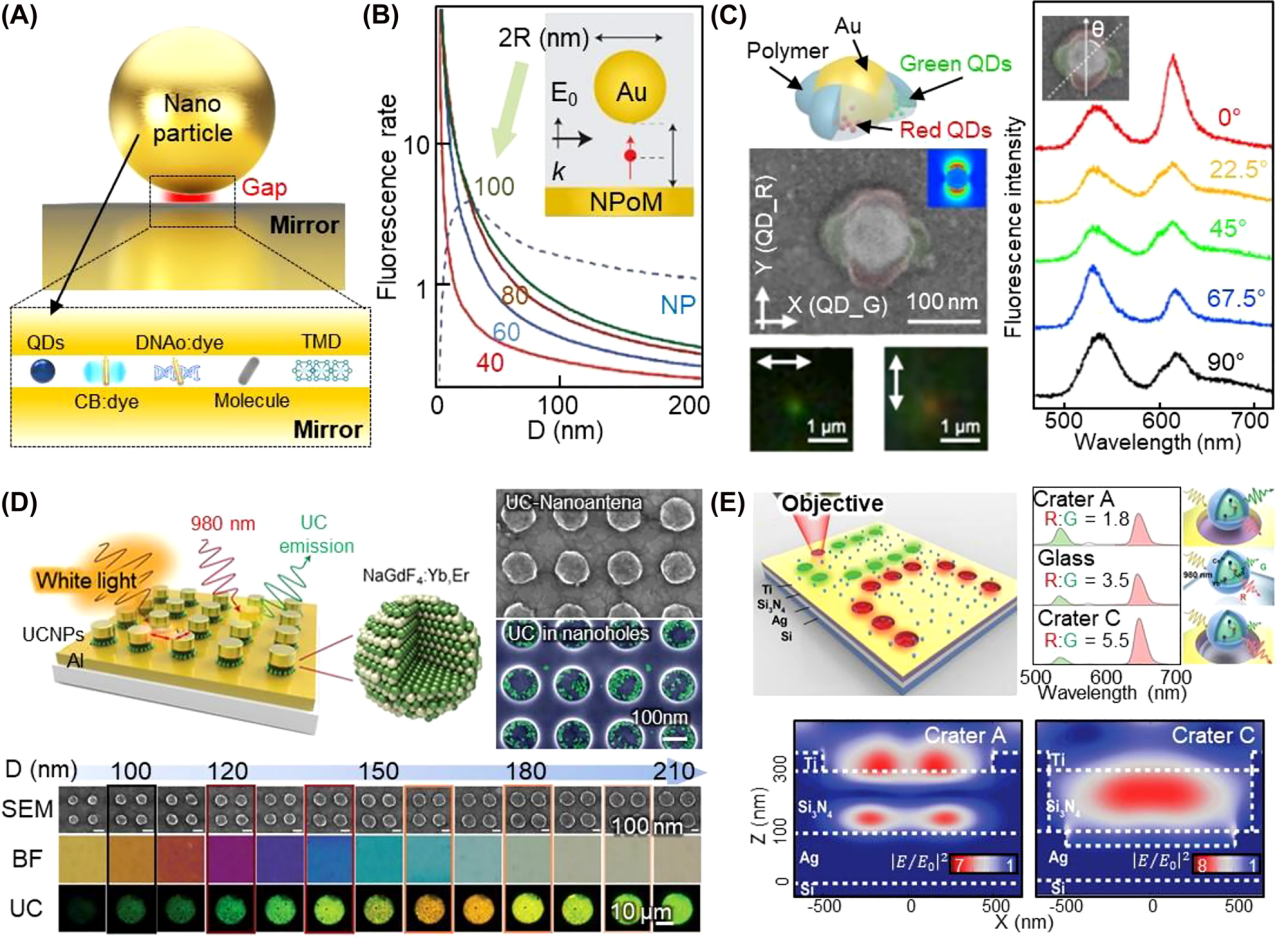
Hybrid plasmonic nanopixels with bandgap nanoparticles for the converted light coloration: (A) plasmonic nanoparticle-on-mirror construct with various emitters in the gap and (B) their associated optical near field enhancement with emitter (reproduced with permission [134], Copyright 2018, American Chemical Society), (C) plasmonic nanoparticle orthogonally decorated with two different types of quantum dots (for red and green emissions) which independently excite the lights depending on the polarization of the light (reproduced with permission [195], Copyright 2015, American Chemical Society), (D) plasmonic nanoparticle-on-mirror construct and (E) nanohole-patterned plasmonic junctions filled with upconversion nanoparticles in their gaps (and holes) (reproduced with permission [191, 192], Copyright 2019, Wiley-VCH and 2019, Wiley-VCH).

The adoption of optical-field enhancement supported by either plasmonic or Mie scatterers is a robust route to amplifying intrinsically low optical signals, such as Raman [166], [167], [168], [169], [170] and nonlinear harmonic scatterings [171], [172], [173], [174], [175]. This approach can also be taken for emitters, such as fluorescent dyes [167, 176], [177], [178], quantum dots [177, 179], [180], [181], [182], [183], 2D materials [166, 184], [185], [186], [187], perovskite [188], and upconversion nanoparticles [189], [190], [191], [192]. If these emitters are positioned near the plasmonic (or Mie) scatterer, they will have modified absorption and emission rates, quantum efficiencies, and radiation patterns under the effect of the optical-field enhancement (Figure 8A) [135]. For example, emitters have been positioned in a ∼5 nm gap of Au nanosphere on an Au mirror construct (Figure 8B). Their spontaneous emission rate is enhanced by a factor of approximately 1000, leading to ultrafast timescales (10 ps) of photon emission (initially ∼10 ns) [135, 177], the quenching suppression, and the strong coupling [193]. However, a meticulous structural design is required as the shape and intensity of the locally enhanced optical field are greatly affected by the size, shape, and optical properties of the plasmonic nanostructures and the relative position of the emitter [134, 135, 194]. In particular, because there is a chance to decrease the emission intensity because of the non-radiative decay caused by the metal (nanoparticle or film) [135, 136, 183], balancing the trade-off between high field enhancements and non-radiative decay is crucial for hybrid plasmonic nanopixels with light-emitting nanoparticles; e.g., see Ref [110, 135] for the underlying physics and functions of the nanoparticle-on-mirror construct in this regard.

We here present an example of hybrid plasmonic nanopixels for dual-color down-conversion emissions (Figure 8C) [195]. The surfaces of 90 nm Au nanoparticles are decorated with eight green (CdSe/ZnS) quantum dots on one axis and eight red quantum dots (CdSe/CdS/ZnS) on another orthogonal axis by embedding them into the polymer matrix. The linear polarization of light (*λ* = 730 nm) then partially enhances the local optical field at the surface of the Au nanoparticle along the axis of the light polarization, such that the selective localization of plasmonic coupling with the quantum dots is possible. Thus, switching between green and red coloration with a single wavelength at the single nanoparticle level can be achieved by rotating the angle of the linear polarization from 0 to 90°.

Upconversion nanoparticles combined with plasmonic nanostructures are another example of hybrid nanopixels (Figure 8D–E) [191, 192]. The 10–20 nm lanthanide upconversion nanoparticles (NaGdF_4_:Yb, Er nanocrystals) are positioned inside the polymer nanoholes (diameter of 130 nm, thickness of 200 nm) patterned on the Al film, covered by an Al nanodisk cap (diameter of 130 nm, thickness of 40 nm, 7.5 nm thick SiO_2_ spacer on the downward side) (top panel in Figure 8D). These nanoparticle-on-mirror constructs generate plasmonic scattering colors based on their gap and lattice dimensions upon the irradiation of white light (bottom panel in Figure 8D) [192]. Interestingly, these colors can be entirely changed when a near-infrared laser (*λ* = 980 nm) is used. This is because the optical near-field enhancement in the gap strongly excites the upconverted colors supported by the upconversion nanoparticles. In contrast, the inverted multilayered nanoholes can be used for this plasmonically driven light upconversion (Figure 8E) [191]. The multilayered Ti-Si_3_N_4_-Ag is drilled by a laser with different intensities (270, 461, 1998 mI cm^−2^) to formulate nanoholes with different depths. These holes are then filled with upconversion nanoparticles (NaYF_4_:Yb/Ho/Ce) having emission wavelengths of 538 and 644 nm. The ratio of red/green (R/G) supported by the upconversion nanoparticle is approximately 3.5 on a cover glass while decaying to 1.8 on the Si_3_N_4_ layer owing to Fabry–Perot resonance. Meanwhile, this ratio can be amplified to 5.5 on the Ag layer when the plasmonic resonance is matched to the wavelength of the red color. These hidden colorations working only at a specific wavelength can be used for imaging-based information encryption and anticounterfeiting (see Section 5.2).

## 5 Emerging applications

The plasmonic and Mie resonance nanopixels above give rise to unique responsive optical dynamics, serving as a new platform for various applications based on coloration, otherwise hard to realize with conventional optoelectronic devices [196]. This section highlights three recent key applications of plasmonic and Mie resonance nanopixels, namely flexible color-changing films and displays (Section 5.1), information encryption and anticounterfeiting (Section 5.2), and active holograms (Section 5.3).

### 5.1 Flexible displays

**Figure 9:**
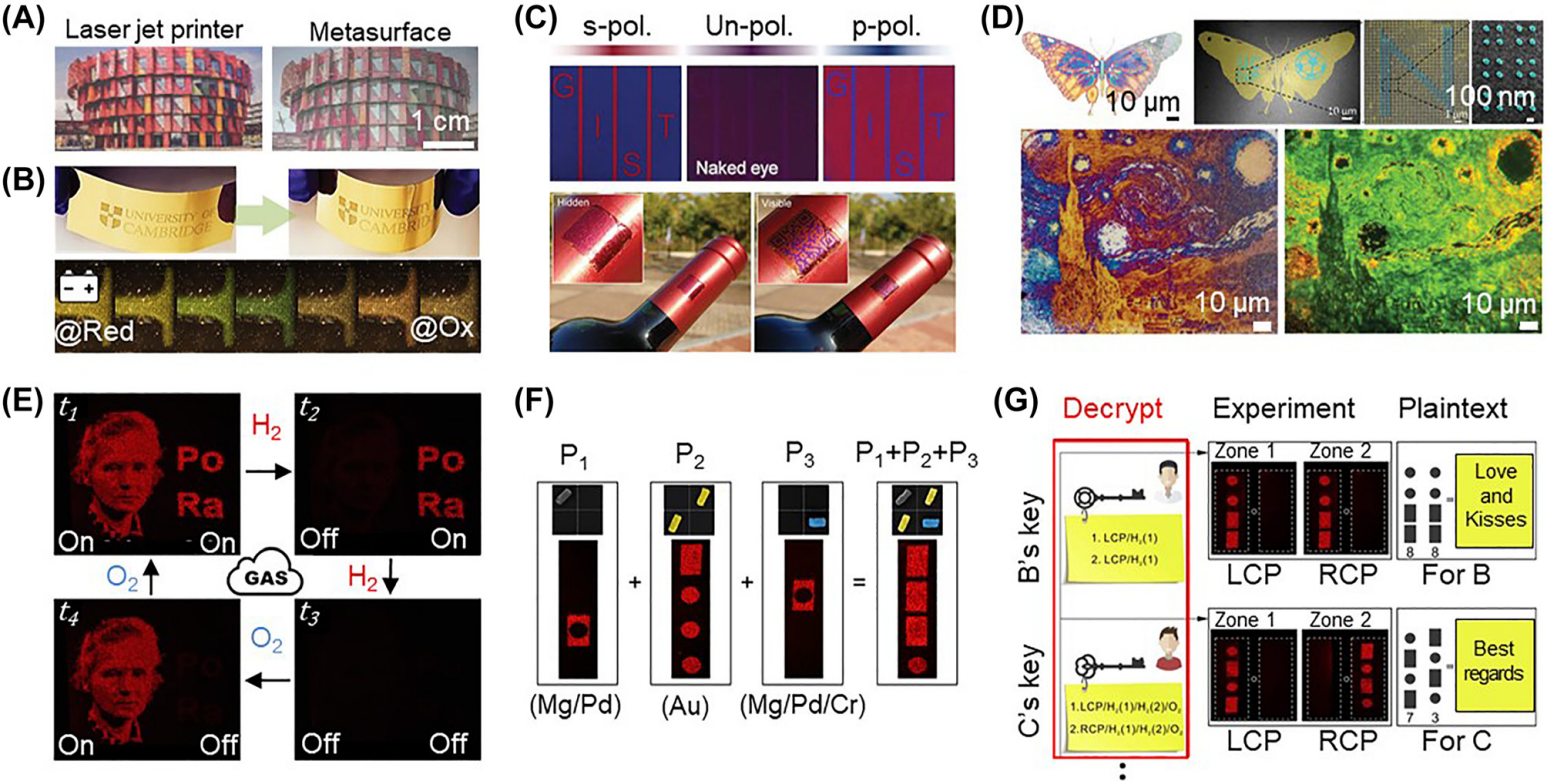
Emerging applications of plasmonic and hybrid nanopixels: (A–B) flexible electrochromic displays using (A) plasmonic nanohole metasurface and (B) plasmonic Au nanoparticle-on-mirror construct filled with conductive polymers (reproduced with permission [125, 198], Copyright 2021, Wiley-VCH and 2020, American Chemical Society), (C–D) information encryption with (C) slanted Ge nanorods for polarization dependent coloration and (D) multilayered plasmonic nanostructures with embedded upconversion nanoparticles for light wavelength dependent coloration (reproduced with permission [198, 199], Copyright 2020, Wiley-VCH and 2019, Wiley-VCH), and (E) active holograms using Mg plasmonic metasurface for (F–G) gas responsive information encryption (reproduced with permission [78], Copyright 2018, American Association for the Advancement of Science).

Flexible and wearable displays demand small optoelectronic devices that provide bright and vivid coloration [3, 8, 12, 21, 125, 197]. Micro- or nano-sized compound semiconductor LEDs [8, 12], quantum-dot LEDs, and organic LEDs [3, 197] have been developed for this use, but they still require technical challenges to be overcome for commercialization. In the case of micro- or nano-sized compound semiconductor LEDs, it is difficult to produce a pixel with a spatial dimension below the micron level owing to the low quantum efficiency, which limits the resolution, whereas quantum-dot LEDs and organic LEDs are difficult to view under sunlight [8, 12, 21]. Owing to their small size and the ambient light-driven coloration, plasmonic nanopixels can readily be adopted for flexible color-changing devices (Figure 9A and B) [125, 198]. For example, 158 nm Cu plasmonic nanoholes have been patterned on an Al mirror (with an Al_2_O_3_ spacer having thickness of 50–150 nm) formed on flexible plastic and shown vivid colors through the broad visible-light range (CIE 1931) (Figure 9A) [198]. An electrochromic polymer (here, polypyrrole or PEDOT:PSS) is coated on the plasmonic (nanohole patterned) film for electrochromic on/off switching. Only a voltage of ±1 V is applied to drive the resonance color switching with an intensity contrast of 90% and switching period of less than 1 s. In contrast with this on/off switching, because PANI intrinsically has large Δn in the visible [108, 110, 125], PANI can be used to tune the scattering colors from red to green, ultimately reducing the pixel size to the nanoscale dimension (<100 nm) (Figure 9B) [125]. The Au nanoparticles encapsulated in the PANI layer are coated on a 15 nm-thick Au film formed on a flexible plastic while still acting as nanopixels to show color dynamics ranging from 580 to 620 nm at the single-nanoparticle level. One further intriguing factor in controlling the color dynamic is the Au mirror [125]. If the thickness of the Au mirror is less than 5 nm, then the light scattered from the nanoparticle at the topside can penetrate the mirror underneath, revealing the color dynamics at the mirror’s backside; i.e., there is active bidirectional coloration. This color-changing film has a power density of only 0.3 mW cm^−2^ and switching at a near video rate (100 Hz), and it is thus promising for low-power-driven display applications. However, the major challenge with this scheme is the generation of the blue color, which might be possible using ultraviolet plasmonic materials, such as Ga [73], Mg [74], and Al [72].

### 5.2 Encryption and anticounterfeiting

In modern society, where the value of personal, corporate, and military information is gradually increasing, safely protecting such valuable information and data against fraudulent and illegal decryption technologies is challenging [14, 200]. One emerging and powerful approach to ensuring the security of this information is using an optical authentication system, which is optical encryption that combines non-replicable physical functions (e.g., material and geometrical information) with optical variables (i.e., the wavelength, intensity, phase, and duration). The ability to selectively switch the color appearance in response to an external stimuli is an excellent characteristic for imaging encryption and anticounterfeiting techniques; e.g., Intaglio Latent Images in a passport can be viewed only at a specific viewing angle or wavelength (Figure 9C,D) [198, 199]. Optical metasurfaces have recently been used in colorful encryption and hidden-image processing with controllable pixelized coloration [78, 198, 201], [202], [203], [204], [205], [206], [207], [208]. The development of functional control in the optical imaging of the nanopixels can provide multiple complex verification procedures, making information inaccessible without the use of predefined passwords [78, 198, 199, 202], [203], [204], [205], [206], [207], [208]. The hidden or encrypted images cannot be identified by the naked eye and only appear in response to the desired external stimuli; e.g., the linearly polarized state (Figure 9C) [199] or specific wavelength and intensity of light (Figure 9D) [192]. For example, an array of slanted Ge nanorods (20–60 nm in length) has been grown on Au thin film to show resonant colors (Figure 9C) [199]. Owing to the nature of the structural anisotropy of the Ge nanorods, polarization-dependent colors can be achieved ranging from 500 to 600 nm along the long and short axes. Therefore, if the nanorods are formed with the hidden pattern image, the colorful pattern can be seen only when the light polarization is aligned along the long or short axis of the nanorods whereas it vanishes under unpolarized light. These patterns can be formed on a curved surface, such as that of a glass wine bottle (bottom panel in Figure 9C), meaning that flexible encryption tapes and cloths can be made. Furthermore, light-driven information encryption and anticounterfeiting techniques are also possible with hybrid plasmonic nanopixels combined with upconversion nanoparticles (Figure 9D) [192]. Plasmonic scattering colors define a colorful image under white light, revealing completely different hidden image patterns only when infrared light is applied, thanks to light upconversion. Technical issues, including optical crosstalk, design complexity, and mass production, remain to be addressed for commercialization, but they are expected to be solved in the near future [14, 200].

### 5.3 Active holograms

Rapid advances in the development of metasurfaces and nanostructures have begun unlocking the huge potential of flat optics [13, 209], [210], [211], [212], [213], but the use of such metasurfaces and nanostructures in holographic imaging applications has so far been limited. They are often static and only show one or two images with limited color dynamics. Thus, individually color addressable nanopixels (or meta-atoms) are required to develop 3D holographic display and optical projection devices [13, 14, 210, 211]. To address this, Mie and plasmonic metasurfaces have been developed for active holograms and 3D displays through the dynamic modulation of the dielectric constant of the color elements (Figure 9E–G) [78]. When the Mg nanopixels are hydrogenated, or the reverse phase transition between the metal and dielectric states occurs, image projection through these nanopixels can be switched between on and off states [78, 155]. When this transition rate is controllable in the manner discussed in Section 3.3 (i.e., by slowing the reaction by partially capping surrounding the nanopixels with nonreactive materials, here Cr), then multiple holographic imaging is possible with nanopixels designed for different reaction rates [78, 155]. In the example shown in Figure 9E, one image plane (showing an image of Marie Curie) is projected with fast-switching nanopixels (having a switching period of 550 s) while the others are projected with slow-switching nanopixels (6000 s). This controllable sequential imaging is beneficial for imaging-based encryptions (Figure 9F and G). The imaging codes are formulated with three different reactive nanopixels made from Mg/Pd (fast switching), Mg/Pd/Cr (slow switching), and Au (nonswitching), as shown in Figure 9F. The nonhydrogenated Au nanopixels always show the same image plane while the other nanopixels appear selectively according to the degree of hydrogenation. Furthermore, a dielectric change in the Mg material affects the phase of the electromagnetic wave, leading to additional encryption complexity (Figure 9G) [78]. As a result, a precise multiple encryption/decryption method can be realized by controlling the phase of the incident light and reactive gas injection (for hydrogenation), but issues such as throughput, stability, and reproducibility need to be addressed for 3D holographic imaging devices [214].

## 6 Conclusions and outlooks

Hybrid nanoscatterers comprising metallic or dielectric nanoparticles show plasmonic or Mie resonant colors, respectively, and can be combined with functional materials that change their optical properties in response to stimuli, such as electrons, light, heat, strain, and gas. They thus ultimately act as responsive photonic nanopixels, allowing us to develop low-power-driven displays and holograms with ultrahigh resolution. This review demonstrates how plasmonic or Mie scattered coloration supported by individual nanopixels can be activated through modulation of one of the resonant parameters; i.e., parameters relating to the geometry and material composition of the nanoparticles and the surrounding optical environment. Depending on the types of responsive materials applied to the nanopixels, the nanopixels function as optical on/off switches or color-tunable pixels operated by the associated external stimuli. We classify three main types of pixel, namely plasmonic and Mie-type pixels and their hybrids with bandgap nanoparticles. They are further sequentially categorized according to three modulation parameters and external stimuli, ranging from electrochromic to mechano- and thermo-chromic systems, and gas reactive coloration. The former plasmonic and Mie resonant nanopixels are mainly operated to generate colors and image planes under white light. In contrast, the later hybrid nanopixels reveal additional (hidden) image planes through the aid of light down- or up-conversion process working only at a specific wavelength. Our classification and summary will help in the development of high-performance nanopixels as they provide information on what types of material composition and geometry of the nanopixels can satisfy the on-demand functional requirements depending on the application. Several important features of plasmonic and Mie scatterers are summarized in Table 1.

**Table 1:** Features of plasmonic and Mie scatterers (ED: electric dipole, EQ: electric quadrupole, MD: magnetic dipole, MQ: magnetic quadrupole).

Specification	Plasmonic scatterer	Mie scatterer	Reference
Dimension	10–100 nm	>100 nm	[17], [18], [19, 47]
Heating (∝ absorption)	High	(Extremely) low	[13, 17, 47, 180, 215], [216], [217]
Resonance mode	ED (+ EQ, ⋯)	ED (+ EQ, ⋯) + MD (+ MQ, ⋯)	[13, 17], [18], [19, 47]
Q-factor	Low (typically < 10)	High	[13, 42, 47, 127, 217, 218]
IC compatibility	Moderate	Good	[14, 133, 216, 219, 220]
Material stability	Low – high (with protection)	High	[17, 127, 133, 221]
Scalability	High	Low (metasurface) – high	[14, 17, 133, 216, 219]

The main advantage of nanopixels is their low-power-driven color dynamics in the visible regime at the single-nanoparticle level under sunlight, and nanopixels thus satisfy the needs for future optoelectronics, ranging from portable, flexible displays (e.g*.*, smart glasses and widows for augmented reality) [133, 153, 218, 222], [223], [224] to outdoor color-changing fashion items and wallpaper [225]. Furthermore, the complex hybridization of the nanopixels continuously expands the application domain, to include active optical encryptions, anticounterfeiting, and holograms. However, despite the great success in developing novel types of the photonic device using plasmonics and Mie resonances, several challenges should be addressed for commercialization. First, the large-area fabrication of active nanopixels with high structural fidelity remains challenging. The nanolithographic means can only produce small prototype devices [226] whereas scalable approaches suffer from low structural reproducibility [110, 125, 227], [228], [229]. Fortunately, recent technical advances in scalable nanofabrication have begun to show promise for complex nanostructuring with high uniformity [70, 226, 230, 231]. Hence, they can potentially accelerate the development of commercially available plasmonic and Mie type color-changing films and devices. Next, the needs for high-end display applications are (at least) high brightness, contrast, and resolution, long-term stability, and low power consumption [3, 5], and there is no single plasmonic or Mie resonance method that satisfies all these requirements. The requirement for video-rate switching was one of the biggest bottlenecks, but it has been met by using the nanoscale dimension of the responsive material within the photonic nanopixels so that the associated overall switching reaction can occur quickly [110, 125, 232, 233]. In particular, the switching speeds of the nanopixels with electrochromic polymers that modulate either the material state of the nanoparticle, $\Delta \epsilon_{r}$ , or the surrounding optical environment, Δn , work at a video rate (50–60 Hz) yet there is permanent damage and deformation of the polymers during the redox process. Therefore, the long-term stability of the display device still needs to be investigated. Such a challenge also applies to the thermo- and mechano-chromic devices used for Δn because there is permanent damage to the devices in both concepts. In contrast, apart from the polarization effect, there seems to be no mechanism of reversible color dynamics based on shape modulation of the nanoparticle, Δχ , and realizing reversible Δχ for plasmonic nanoparticles thus remains challenging. Although there should be further problems specific to each concept of device as briefly discussed above, we stress the common issue that, to the best of our knowledge, there is thus far no pixelized full-color display using either plasmonic or Mie resonance, which should be addressed for commercial application [38, 203, 214, 216, 218, 234].
